# A new LNC89/LNC60-Col11a2 axis revealed by whole-transcriptome analysis may be associated with goiters related to excess iodine nutrition

**DOI:** 10.3389/fendo.2024.1407859

**Published:** 2024-10-31

**Authors:** Guanying Nie, Shuang Li, Wei Zhang, Fangang Meng, Zixuan Ru, Jiahui Li, Dianjun Sun, Ming Li

**Affiliations:** ^1^ Key Lab of Etiology and Epidemiology, National Health Commission & Education Bureau of Heilongjiang Province (23618504), Key Laboratory of Trace Elements and Human Health, Center for Endemic Disease Control, Chinese Center for Disease Control and Prevention, Harbin Medical University, Harbin, China; ^2^ Department of Endocrinology and Metabolism, The Second Affiliated Hospital of Harbin Medical University, Harbin, China

**Keywords:** iodine excess, thyroid, goiter, Col11a2, lncRNA

## Abstract

Goiter related to excessive iodine nutrition remains a significant public health issue in some countries. There has been no reported study on long noncoding RNAs (lncRNAs) related to goiters. In this study, goiter was induced by drinking water with excess iodine for 10 or 20 weeks in Kunming mice. Whole transcriptome sequencing results showed that LNC89 expression increased in mice goiter tissues compared to normal thyroid tissues and higher in 20 weeks goiter tissues than in 10 weeks goiter tissues, which were identified by qRT−PCR. Cooperate with human-mouse homologous gene conversion, a new LNC89/LNC60-Col11a2 axis was predicted by LncTar and expression correlation analysis based on whole transcriptome sequencing results. Increased Col11a2 expression was also identified by qRT−PCR and Western blot in the mice goiter tissues. In the human normal thyroid cell line Nthy-ori-3 treated with KI0_3_, LNC60 and Col11a2 expression increased with promoted cell viability, which were reversed by siLNC60 treatment. Furthermore, LNC60 and Col11a2 mRNA levels were found increased in peripheral blood of nodular goiter patients from high water iodine areas of China and have high diagnostic values for nodular goiter while AUC of LNC60 and Col11a2 are 89.97% and 84.85%, respectively. In conclusion, the novel LNC89/LNC60-Col11a2 axis may be involved in the progression of goiter related to iodine excess, providing potential biomarkers and therapeutic targets in the future.

## Introduction

Water with high iodine content and iodine rich foods are two major factors related to iodine excess worldwide. In addition to Japan and Korea, which have iodine rich foods, high-water-iodine areas are primarily found in 13 countries and regions worldwide (1). According to China’s 2017 water iodine monitoring results, 51 administrative counties including 25,317 administrative villages still had high water iodine levels exceeding 100 μg/L, affecting 40.65 million people (2). Although iodized salt supply has been halted, over 80% of surveyed individuals in high-water-iodine areas had a median urinary iodine level above 300 μg/L, indicating a risk of excessive iodine intake (3, 4).

Iodine excess so far was found related to various thyroid diseases, including goiter (5),.Goiter prevalence was 6.3% to 11% in the areas with high water iodine levels, while goiter prevalence increased with water and urinary iodine levels (4, 6). Various theories have been proposed to explain the development of goiter, including the iodine blocking effect (Wolff−Chaikoff effect), inhibition of hormone secretion due to gland storage, inhibition of the sodium-iodine symporter (NIS), intrathyroidal redistribution of organic iodine, colloid retention, and others (5, 7). However, there is no consensus on the exact mechanisms behind goiter, and further research is needed.

The primary type of goiter caused by iodine excess is colloid goiter, also known as diffuse goiter (8, 9), which was not noticed at the early stage. However, as the damage gets worse, the thyroid volume gradually increases, possibly leading to breathing difficulties and difficulty swallowing. Patients may need treatments like radio frequency ablation, laser therapy, or surgery. These treatments can burden patients and harm their thyroid function permanently. Additionally, due to goiter can increase the risk of thyroid nodules and thyroid cancer, goiter patients may require regular ultrasound exams. Therefore, it is urgent to elucidate its mechanism and develop new therapeutic methods.

Long noncoding RNAs (lncRNAs) have emerged as a novel type of biomarker and play a crucial role in the diagnosis and treatment of various diseases. LncRNAs may be involved in the regulation of gene expression through transcription, chromatin modification, posttranscriptional regulation, and translation (10–12). To date, numerous lncRNAs have been discovered in thyroid cancer, such as FOXD3-AS1, BRM, HOTAIR, and MALAT1. However, there was no report on lncRNAs related to other thyroid disease (13–15).

In this study, based on the whole transcriptome sequencing, we hope to find a new lncRNA−mRNA co-expression network related to goiter and identify new molecular markers for goiter diagnosis and therapy.

## Method

### Animals

Eighty female Kunming mice (18 ± 4 grams and 4 weeks of age) were purchased from Vital River Laboratories Animal Technology Co., Ltd. (Beijing, China) and raised in specific pathogen free (SPF) conditions with constant temperature and humidity (temperature 23 ± 1°C, humidity 45% ± 5%, 12-hour light, 12-hour dark cycle). The research committee certified that the study subjects were healthy and fit for the experiment. Five mice per cage were allowed free access to food, water, and commercial SPF mouse maintenance feed. After a week of acclimation to the diet, all mice were randomly assigned to four groups: Control 1, Control 2, Treatment 1 and Treatment 2. Control 1 and Treatment 1 were fed for 10 weeks, and Control 2 and Treatment 2 were fed for 20 weeks. Potassium iodate (KIO_3_) solution was dissolved in double distilled water (DDW). DDW with 50 μg/L iodine is for control groups and 300 μg/L iodine is for the treatment groups. The chow iodine content was 305 ± 142 μg/kg, and approximately 5-8 g of food and 5-10 ml of water per mouse were considered in a day. The estimated iodine intake of the mice in the control groups was 1.5 μg/d and that of the mice in the treatment groups was above 4 μg/d. The committee of Harbin Medical University approved the animal experiment protocols. Experimental protocols and animal welfare guidelines were in accordance with the Care and Use of Laboratory Animals, National Institutes of Health (NIH). Ensure that the animal is provided with a proper living environment, behaves normally, has no irritable behavior, and the high iodine dose is not super high, and that gnawing stick is equipped. The animals were euthanized at the end of the study to ensure no additional suffering.

### Sample preparation

During the feeding period, mice were observed daily, and their weights were measured weekly. Before euthanasia, 24-hour urine samples were collected from mice using metabolic cages. Urine was filtered through a 0.22 μm filter, centrifuged (3000×g, 5 minutes, room temperature), and then stored at -20°C for measuring urinary iodine levels. Mice were anesthetized with pentobarbital, cardiac blood was collected and centrifuged (4000×g, 15 minutes, room temperature), and the serum was stored at -80°C for measuring thyroid function and serum iodine levels. After euthanasia, the left thyroid lobes of six mice from each group were fixed in 2.5% glutaraldehyde (Sigma−Aldrich, USA) for TEM electron microscopy, and the corresponding right thyroid lobes were fixed in 4% paraformaldehyde (Biosharp, China) for paraffin section preparation. Fourteen mouse thyroid samples were snap-frozen in liquid nitrogen and stored at -80°C. Among them, three randomly selected thyroid tissue samples from the control groups and three randomly selected goiter tissues from treatment groups were used for whole transcriptome sequencing, while the remaining samples were used for real-time quantitative PCR (qRT−PCR) and Western blot (WB) experiments.

### Measurement of urinary iodine, serum iodine, iodine in feed, and thyroid function (TSH and FT4)

Urinary iodine (UI), serum iodine (SI), and iodine in the feed were measured according to the standards WS/T 107.1-2016, WS/T 572-2017, WS 302-2008 and GB 5009.267-2020 (16–19) Thyroid-stimulating hormone (TSH) and free thyroxine (FT4) levels were measured using enzyme-linked immunosorbent assay (ELISA) kits (Kenuodi, China CK-E20360 and CK-E20383) in 10ul serum according to the kit protocol. The detection range of TSH is 0.75 to 24mU/L with accuracy 0.1mU/L, of FT4 is 1.25 to 40 pmol/L with the accuracy 0.1pmol/L.

### Transmission electron microscopy

Samples were prepared and dehydrated by laboratory personnel according to standard procedures and observed under a microscope (Hitachi 7700, Japan). Water is extracted from the tissues by passing them through the graded alcohol as 30%, 50%, 70%, 80%, 90%, 95%, absolute alcohol, and then absolute acetone.

### Hematoxylin and eosin staining and immunohistochemistry analysis

Tissues were embedded and cut into 4 μm sections. After deparaffinization, H&E staining and immunohistochemistry were performed according to standard methods. After section preparation, images were captured using a BX 53 microscope (Olympus, Japan).

### RNA extraction and RNA sequencing

Total RNA was extracted using TRIzol reagent (TaKaRa, Japan), and RNA concentration and quality were detected using a NanoDrop spectrophotometer (NanoDrop Technologies, USA) and Invitrogen Qubit 3.0. A spectrophotometer (Thermo Fisher Scientific, USA) was used. RNA-seq was performed on the Illumina HiSeq 2500 platform (Genesky Biotechnologies, Inc., Shanghai, China) using a 2×150 bp paired-end sequencing strategy. All differentially expressed genes were selected based on the criteria of fold change ∣log_2_FC∣ > 1 and *P* < 0.05.

### Human-mouse homologous gene conversion and Lnc RNA target mRNA analysis

The NONCODE website (http://www.noncode.org/) was accessed to retrieve gene sequences. NCBI BLAST was entered for sequence alignment to select highly homologous human lncRNAs. We used LncTar software for predicting RNA targets of lncRNAs (20). For a pair of lncRNA and mRNA which are predicted to have interaction, they also must have a strong co-expression. We set a minimum required Pearson correlation coefficient (PCC) of 0.5 between them for this purpose.

### Quantitative real-time PCR

Total RNA was reverse-transcribed to obtain cDNA using the RT−PCR First-Strand cDNA Synthesis Kit (Roche, USA). Quantitative real-time PCR was performed using the SYBR method (Roche, USA), and gene expression in triplicate experiments was compared using the 2^-ΔΔCt^ method. Mouse β-Actin was used as the internal reference, and the primers sequence can be found in **Supplementary Table 1.**

### Western blot

Total protein was extracted from the tissues using radioimmunoprecipitation assay buffer (RIPA) buffer (Beyotime, China) and quantified using a bicinchoninic acid (BCA) BCA protein assay kit (Beyotime, China). Twenty micrograms of protein from each sample were separated on a 15% polyacrylamide gel through electrophoresis and transferred to obtain immunoblot bands. Finally, the bands were visualized using a chemiluminescence imaging system (Tanon, China). The grayscale values of the bands were quantitatively analyzed using ImageJ software (NIH, Bethesda). Primary antibodies are rabbit anti-mouse Col11a2 antibody (YT1009, 1:500, Immunoway, China) and mouse anti-mouse β-actin antibody (TA811000, 1:2000, OriGene, USA) while mouse and rabbit second antibodies from Abbkine (A25012, 1:10000, China) and CST (14708, 1:2000, USA).

### Cell line

The Nthy-ori-3-1 cell line is an immortalized human normal thyroid cell line provided by Professor Qiao Hong’s research group in the Department of Endocrinology at the Second Affiliated Hospital of Harbin Medical University. The cells were authenticated using short tandem repeat (STR) analysis. The cells were cultured at 37°C with 5% CO_2_ in complete medium consisting of 10% fetal bovine serum (Procell, China), 2% penicillin−streptomycin (Solarbio, China), and RPMI-1640 medium (Gibco, USA).

### Cell viability detection

Cells were seeded in 96-well plates at a density of 3000 cells per well and cultured overnight in complete medium. Afterward, they were treated with different concentrations of potassium iodate for 24, 48, 72, and 96 hours. Cell viability was assessed using the Cell Counting Kit-8 (CCK-8, Biosharp) and the ethynyl deoxyuridine (EdU, RiboBio) assay, following the manufacturer’s protocols. We investigated the influence of different iodine concentrations (0, 10^-3^ M, 10^-5^ M, 5×10^-6^ M, and 10^-7^ M) on cell viability in Nthy-ori-3 thyroid cells. Nthy-ori-3 cells were cultured to attain 70-80% confluency before being subjected to iodine treatments for 24 hours. An untreated control group was maintained for comparison. Subsequently, EdU (10 µM) was introduced to the cells during the active cell profiling phase. Following cell harvesting, fixation with 4% paraformaldehyde, permeabilization with 0.3% Triton X-100, and Click reaction using Ruibo’s EdU Click-iT™ kit, cell nuclei were stained with 4’,6-diamidino-2-phenylindole (DAPI). Microscopy and flow cytometry analyses were conducted to quantify EdU-positive cells, providing insights into the impact of iodine concentration on cell profiling in Nthy-ori-3 cells. Statistical analysis was performed to determine the significance of the observed effects.

### Cell transfection experiment

We used Lipofectamine™ 3000 transfection reagent (Thermo Fisher) to transfect LNC60 siRNA into Nthy-ori-3 thyroid cells according to the manufacturer’s protocol. The synthesis of siRNA targeting LNC60 (5’- CACUCAGGCUGCAAGCAGUTT -3’ and 5’- ACUGCUUGCAGCCUGAGUGTT -3’) and nontargeting controls (5’- UUCUCCGAACGUGUCACGUTT -3’ and 5’- ACGUGACACGUUCGGAGAATT -3’) were performed by Anhui Universal Biotech Co., Ltd.

### Case control study of the nodular goiter patients

According to the national standards (GB 16005-2009 and GB/T 19380-2016), the areas with median water iodine (MWI) levels more than 100μg/L were defined as high water iodine areas. From June 2019 to January 2020, an epidemiological survey was carried out in the towns of Malingang, Shiji, Gucheng and Wanfu of Heze city, Shandong province, where the MWI was more than 100μg/L according to the 2017 Chinese national report on water iodine distribution at township level. A total of 1344 people were recruited, including 315 males and 1029 females age 18 to 70 years old. After ultrasound examination, male with thyroid volume greater than or equal to 23ml and female with thyroid volume greater than or equal to 18ml were diagnosed as goiter. Finally, we got 26 patients with goiter, and 26 healthy controls were selected according to age (± 3 years) and sex, 1:1 matching. In these survey areas, the supply of iodized salt was stopped in 2009, therefore the high iodine content in local water resources can reflect the excess iodine nutrition of local residents. Population with other thyroid diseases, disabilities, mental disorders, abnormally low autoimmune function, have took thyroid disease-related drugs, such as methimazole, propylthiouracil, levothyroxine tablets, and so on, have pregnancy or with incomplete research data, such as missing blood or demographic data were excluded from both cases and controls, and informed consent was signed. When the subjects were fasting, 15ml of venous blood was collected by disposable vacuum sampling, and centrifuged at 20-30°C (3000 RPM, 10 minutes). Serum was collected and stored at -80°C in the laboratory for RNA extraction. cDNA synthesis and qRT−PCR detection of LNC 60 and human Col11a2 were performed as above. Human β-Actin was used as the internal reference, and the primers sequence can be found in **Supplementary Table 1.**

### Statistical analysis

Statistical analysis was performed using R software (version 4.3.1). The data normality distribution was validated using the Kolmogorov−Smirnov test. For normally distributed data (such as body weight, thyroid organ coefficient), t tests was used, and data are represented as the mean ± standard deviation. For nonparametric tests, data are represented as the median with interquartile range (IQR) such as serum iodine, urinary iodine, FT4, TSH, and Mann−Whitney U test was used for comparison between groups. A bilateral test was used, with *P* value less than 0.05 indicating statistical significance. Evaluation of diagnostic value depends on receiver operating characteristic (ROC) curve. When the area under the curve (AUC) >0.7 with *P*<0.05, it was considered to have diagnostic values. Data visualization was performed using R packages, including the ggplot2 package for scatter plots, box plots, and bubble plots and the ggVennDiagram package for Venn diagrams.

## Results

### Establishment of the goiter mouse model

At 10 weeks, the goiter group of mice had urinary iodine and serum iodine levels of 569.40 μg/L and 63.13 μg/L, respectively, which were significantly higher than those of the control group at the same period, which had levels of 171.60 μg/L and 26.76 μg/L (P<0.01; P<0.001). At 20 weeks, the high-iodine group of mice had urinary iodine and serum iodine levels of 312.5 μg/L and 62.83 μg/L, respectively, which were significantly higher than those of the control group at the same period, which had levels of 186.90 μg/L and 27.65 μg/L (P<0.01; P<0.001). At 10 weeks, the thyroid weight and organ coefficient of the high-iodine group of mice were 6.93 ± 1.76 mg and 0.171‰, respectively, which were significantly higher than those of the control group at the same period, which were 4.92 ± 2.95 mg and 0.09‰. At 20 weeks, the thyroid weight and organ coefficient of the high-iodine group of mice were 7.54 ± 2.78 mg and 0.163‰, respectively, which were significantly higher than those of the control group at the same period, which were 5.00 ± 1.02 mg and 0.101% (**Table 1**).

**Table 1:** Detection of serum iodine and thyroid function in mice.

Period(Weeks)	Group	Serum Iodine	Urine Iodine	FT4	TSH	Weight	Thyroid Weight	Organ Ratio
		**(μg/L)**	**(μg/L)**	**(pmol/L)**	**(mU/L)**	**(g)**	**(mg)**	**(‰)**
10	Treatment1	63.13(55.76)a**	569.40(78.10)a***	21.32(1.78)	3.18(0.74)	43.59 ± 3.20	6.93 ± 1.76a*	0.171(0.067)
10	Control1	26.76(14.70)	171.60(114.10)	21.87(1.04)	3.11(0.50)	43.67 ± 3.94	4.92 ± 2.95	0.09(0.0592)
20	Treatment2	62.83(33.43)b***	312.50(84.40)b***	22.07(2.58)	3.44(0.96)b*	42.15 ± 5.57	7.54 ± 2.78b***	0.163(0.096)b***
20	Control2	27.65(13.80)	186.90(112.00)	21.87(1.05)	3.10(0.50)	44.30 ± 6.18	5.00 ± 1.02	0.1011(0.044)

a compared with Control1. b compared with Control2. ^**^ *P*<0.01^. ***^ *P*<0.001. *Mann−Whitney U* test.

### Histological changes in the thyroid tissues of goiter mouse model

**Figure 1:**
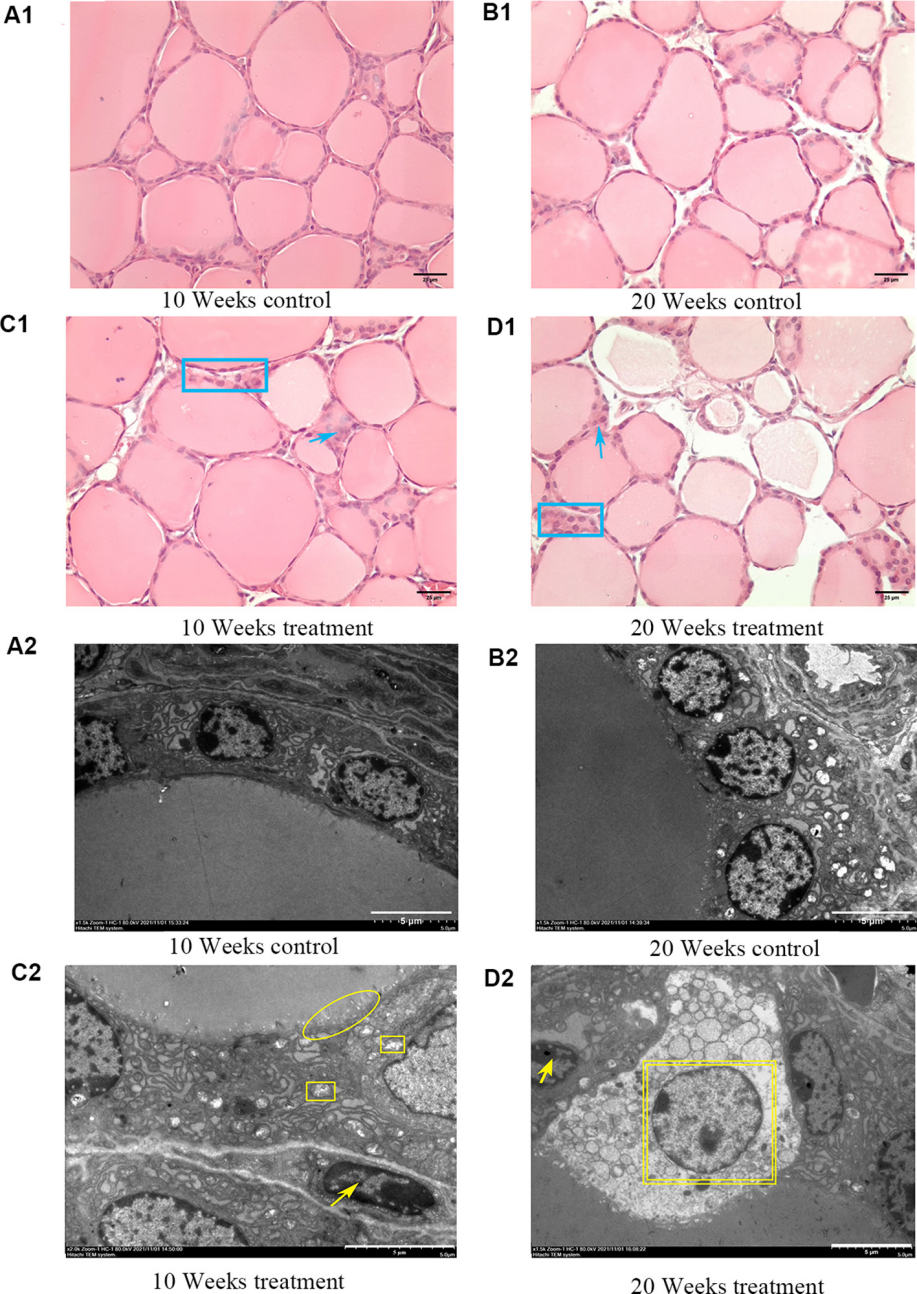
Pathological and TEM ultrastructural changes in thyroid of mice caused by iodine excess. The top image shows thyroid structure by HE staining. **(A1)** 10 weeks control group. **(B1)** 20 weeks control group. **(C1)** 10 weeks treatmen1 group. **(D1)** 20 weeks treatment group. Scale bar = 25 μm. Blue rectangle: nuclei flat with different shapes or overlapping. Blue arrows: inflammatory response. Blue oval: follicular hyperplasia, follicular capsule thinning or even disappearance. The bottom image shows the ultrastructure of the thyroid glands by TEM. Scale bars = 5 μm. **(A2)** 10 weeks control1 group. **(B2)** 20 weeks control group. **(C2)** 10 weeks treatmen1 group. **(D2)** 20 weeks treatment group. Scale bar = 5 μm. Yellow oval: sparse microvilli with or without microvilli ciliation. Yellow square: mitochondrial swelling, yellow arrow: nuclear condensation. Yellow double-layer square: necrotic scar bar=5 μm.

At 10 weeks and 20 weeks, the thyroid follicles of the control group (**Figures 1A1, B1**) mice were rich in colloid, and the thyroid epithelial cells had a cubic or low cubic shape with round cell nuclei and regular arrangement. Excess iodine can cause changes in the pathological structure of the thyroid. Compared to the control group, the 10 weeks high-iodine goiter group (**Figure 1C1**) exhibited enlarged follicles, increased colloid, flattened cell nuclei, and a small amount of inflammatory reactions in the HE staining. In addition to the pathological changes seen in the 10-week goiter group, the 20-week high-iodine goiter group (**Figure 1D1**) showed more severe pathological structural changes, such as empty follicles and thinning or disappearance of the follicle basement membrane. TEM results showed that the thyroid ultrastructure in the control group at 10 weeks and 20 weeks (**Figures 1A2, B2**) was normal, with round and smooth cell nuclei, deep colloid, normal morphology and structure of mitochondria, and endoplasmic reticulum and neat arrangement of microvilli. Compared to the 10-week control group, TEM results in the goiter group at the same time showed ultrastructural changes in the thyroid, including flattened nuclei, swollen mitochondria in thyroid epithelial cells, sparse microvilli, shallower colloid, and slight nuclear condensation (**Figure 1C2**). In addition to the ultrastructural changes observed in the 10-week high-iodine group, the 20-week high-iodine goiter group showed more severe cell apoptosis and necrosis (**Figure 1D2**).

### Thyroid function analysis of the goiter mouse model

At 10 weeks, the levels of TSH and FT4 in the high-iodine goiter group of mice were 3.18 mU/L and 21.32 pmol/L, respectively, slightly higher than those in the control group (3.11 mU/L and 21.87 pmol/L), but the difference was not statistically significant. At 20 weeks, TSH in the high-iodine goiter group of mice was significantly higher than that in the control group (3.44 mU/L and 3.10 mU/L) (*P*<0.05); FT4 was slightly higher than that in the control group (22.07 pmol/L and 21.87 pmol/L), but the difference was not statistically significant (**Table 1**).

### Differentially expressed lncRNAs were revealed by whole transcriptome sequencing in the goiter mouse model

**Figure 2:**
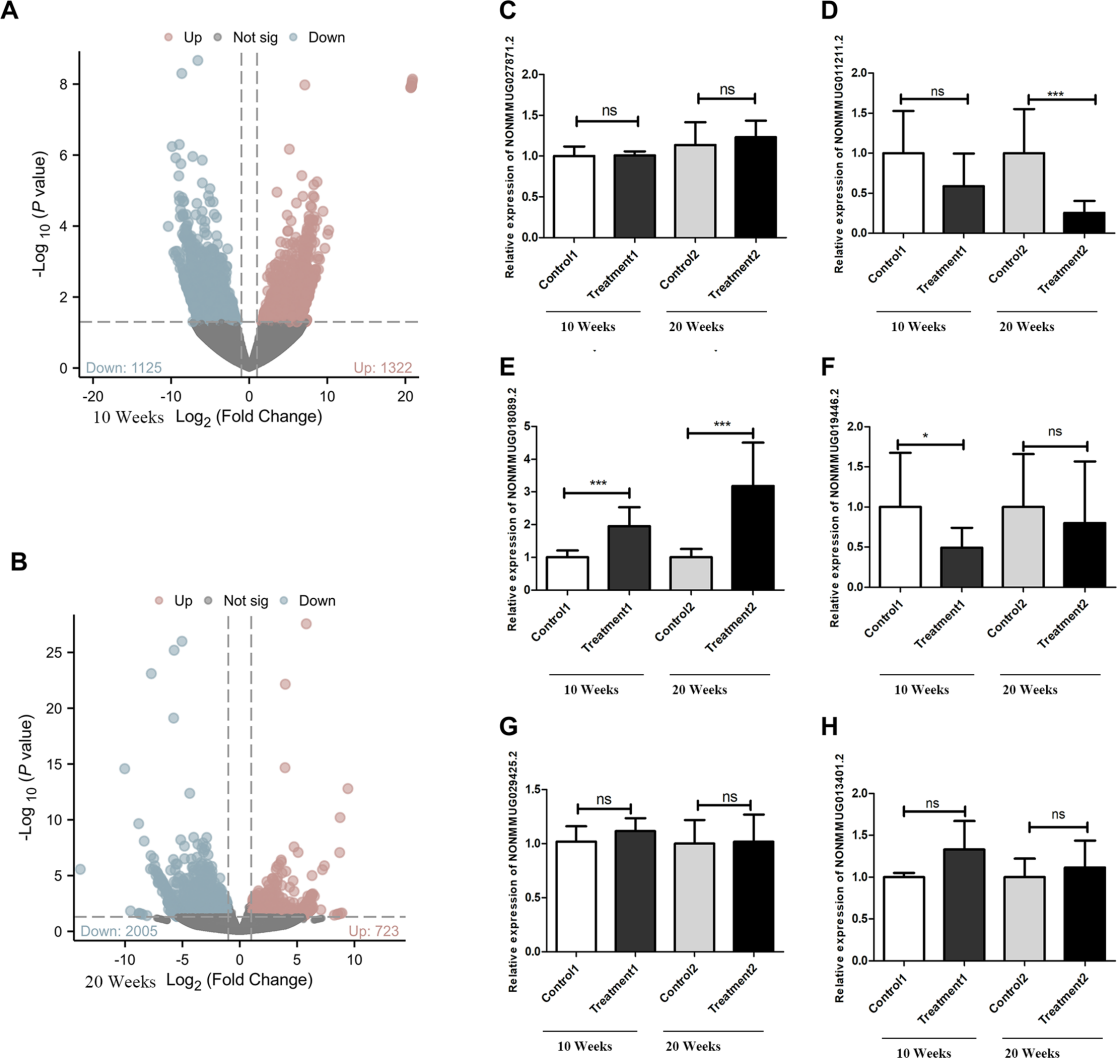
Differentially expressed lncRNAs and verification with qRT−PCR. **(A)** There were 2728 differentially expressed lncRNAs between the 10 weeks control group and treatment groups, 2005 of which were downregulated DElncRNAs and 723 of which were upregulated DElncRNAs, as shown by the volcano plot. **(B)** There were 2447 differentially expressed lncRNAs between the 20 weeks control group and Treatment groups, 1125 of which were downregulated DElncRNAs and 1322 of which were upregulated DElncRNAs, as shown by the volcano plot. **(C)** NONMMUG027871.2 expression level has no difference between treatment groups and control groups. **(D)** NONMMUG011211.2 expression decreased in both treatment groups, but only statistically significant in 20 weeks. **(E)** NONMMUG018089.2 expression levels increased in both treatment groups, with statistically significant. **(F)** NONMMUG019446. 2 expression levels decreased in both treatment groups, but only statistically significant in 10 weeks. **(G)** NONMMUG029425.2 expression has no difference between treatment groups and control groups. **(H)** NONMMUG013401.2 increased in both the treatment groups, but with no statistically significant. *P <0.05. ***P <0.001.

Compared to the control group at 10 weeks, the goiter mice had 723 upregulated differential lncRNAs and 2005 downregulated lncRNAs (**Figure 2A**). Compared to the control group at 20 weeks, the high-iodine group of mice had 1322 upregulated differential lncRNAs and 1125 downregulated differential lncRNAs (**Figure 2B**). We chose differentiated expressed lncRNAs, whose BaseMean are more than 50, the absolute values of log2Fold Chage are more than three in the 20 weeks goiter, and their log2Fold Chage are more than that in the 10 weeks Goiter. We got seven up regulated lncRNA and four down regulated lncRNA, and only six lncRNA can be designed qualified primers, as shown in **Table 2** and **Figure 2**.

**Table 2:** Differentially expressed lncRNA meeting the screening criteria.

ID	Base Mean	log2FC	P	Type
NONMMUG019446.2	86.15498009	-5.021611926	8.86177E-06	Down
NONMMUG013401.2	54.65454322	-4.072693926	0.002492893	Down
NONMMUG011211.2	79.19357743	-3.602198857	0.012952995	Down
NONMMUG029425.2	147.8639377	3.147507597	0.006733359	Up
NONMMUG018089.2	106.6304867	5.194913509	0.006409145	Up
NONMMUG027871.2	29.88260751	3.436631048	0.001566769	Up

### Validation of differential lncRNAs by qRT-PCR in the goiter mouse model

qRT-PCR results showed that the expression of LNCNONMMUG019446.2 (LNC19) was significantly downregulated in 10-week goiter tissues, but not in the 20-week goiter tissues (**Figure 2F**). The expression of NONMMUG011211.2 (LNC11) in the 20-week goiter tissues was significantly downregulated, but not in the 10-week goiter tissues (**Figure 2D**). The expression of LNC NONMMUG018089.2 (LNC89) was significantly upregulated both in the 10-week and 20-week goiter tissues, and the increase was more pronounced in the 20-week goiter group compared to the 10-week high-iodine group (**Figure 2E**). Analysis of the nucleotide sequences of these lncRNAs revealed that only LNC89 had highly homologous human lncRNAs, with the transcript ID being NONHSAT207060.1 (LNC60).

### Construction and validation of the LNC89-Col11a2 regulatory axis

Subsequently, we screened differential expressed genes in the sequencing results, and then, based on correlation analysis, coexpressed mRNAs of LNC89 were identified, and this study selected Col11a2, which had a correlation coefficient exceeding 0.9 with LNC89. The correlation results (R=0.93, P<0.05) are shown in **Table 3**.

**Table 3:** co-expressed mRNA of NONMMUG018089.2.

lncRNA	lncRNA log_2_FC	lncRNA Regulation	mRNA	mRNA log_2_FC	mRNA Regulation	Cor
NONMMUG018089.2	5.19	Up	H2-Ke6	-1.20	Down	-0.81
NONMMUG018089.2	5.19	Up	Col11a2	1.31	Up	0.93**

**Figure 3:**
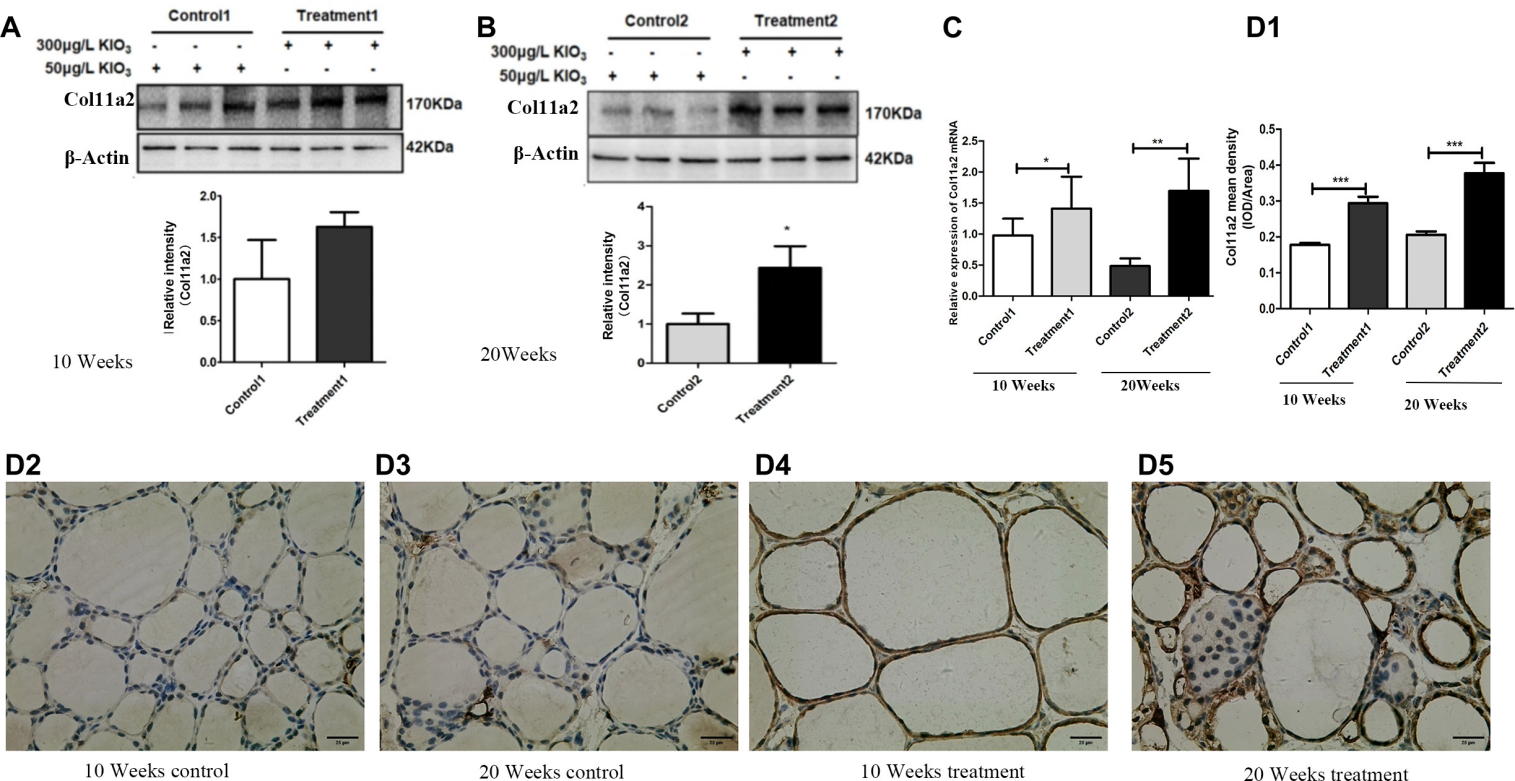
Col11a2 mRNA and protein expression in goiter mouse model. **(A)** Col11a2 protein expression levels increased in the 10 weeks treatment group compared with the 10 weeks control group by western blotting. **(B)** Col11a2 protein expression levels increased in the 20 weeks treatment group compared with the 20 weeks control group by western blotting. **(C)** Col11a2 expression mRNA levels increased in both treatment groups detected by qRT−PCR. **(D)** IHC result. (D1) Quantitative analysis of IHC results. Col11a2 expression levels increased in the 10 weeks treatment 1 group compared with 10 weeks control group. Col11a2 expression levels increased in the 20 weeks treatment group compared with the 20 weeks control group. (D2) 10 weeks control group. (D3) 20 weeks control. (D4) 10 weeks treatment. (D5) 20 weeks treatment group. Scale bar = 25 μm. *P <0.05, **P <0.01. ***P <0.001.

WB results showed that compared to that in the 10-week control group, Col11a2 protein expression in the 10-week goiter tissues of mice was increased, but the difference was not statistically significant (**Figure 3A**). Compared to that in the 20-week control group, Col11a2 protein expression in the 20-week goiter tissues was significantly increased (**Figure 3B**). The qRT−PCR results showed that the Col11a2 mRNA was significantly increased both in the 10-week and 20-week goiter tissues (**Figure 3C**). IHC results showed that compared to the control group, Col11a2 in the 10-week and 20-week goiter tissues displayed significant brown staining (**Figures 3D2**-**5**), and the brown staining in the 20-week goiter tissues was more pronounced than that in the 10-week group. The integral optical density values (IOD) of Col11a2 immunohistochemistry in the goiter tissues were significantly higher than that in the control group (**Figure 3D1**).

**Figure 4:**
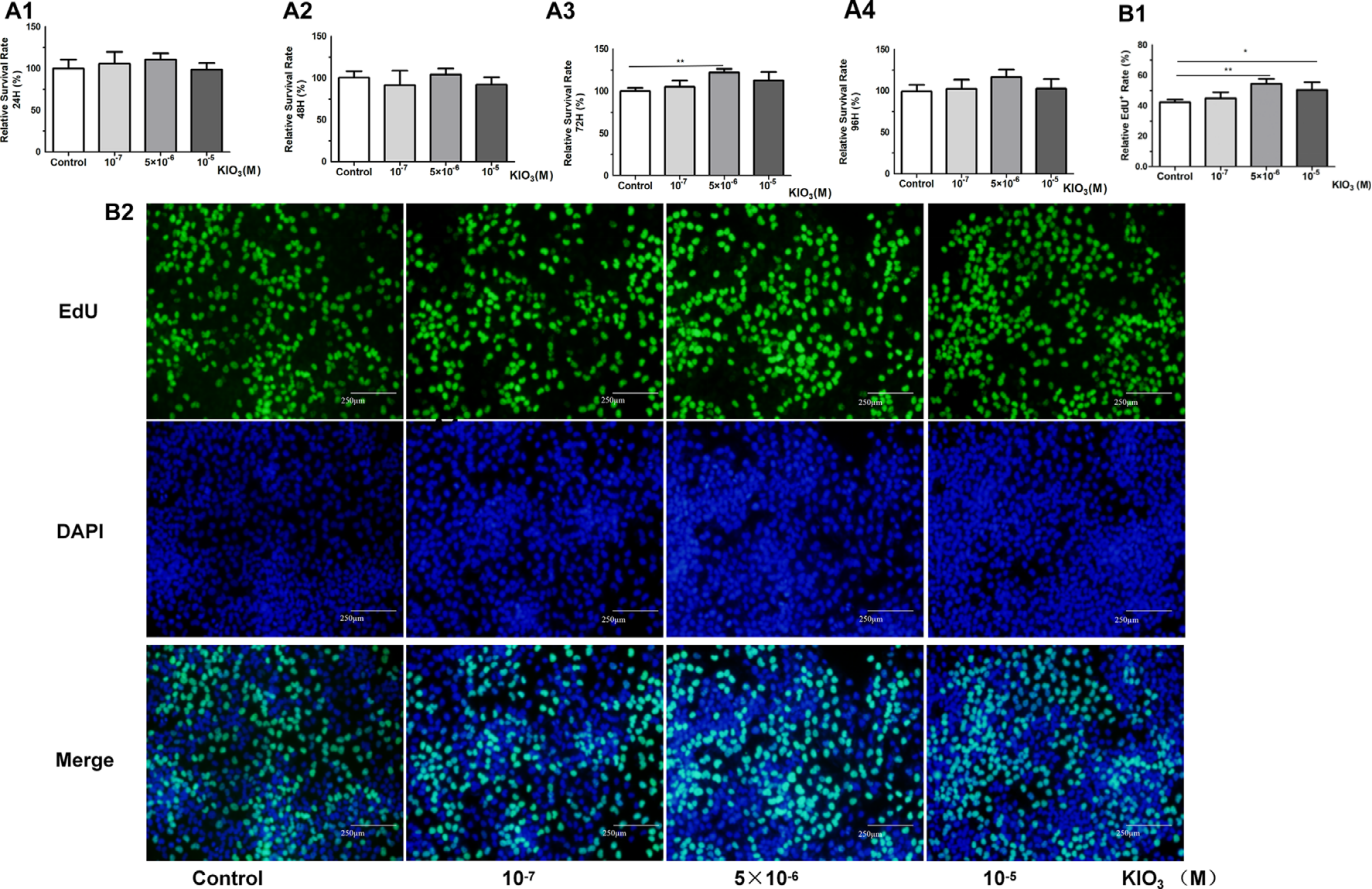
CCK8 and EdU assays were used to evaluate the cell viability of Nthy-ori-3 cells treated with different iodine concentrations. Analysis of cell viability using the CCK8 assay. **(A1)** After 24 hours of treatment, no significant cell viability promotion was observed. **(A2)** After 48 hours of treatment, no significant cell viability promotion was observed. **(A3)** After 72 h of 5×10^-6^ M KIO_3_ treatment, cell viability was promoted. **(A4)** After 96 h of treatment, no significant cell viability promotion was observed. **(B1)** After 72 h of 5×10^-6^ M KIO_3_ treatment, a significant viability promotion in EdU was observed. **(B2)** The lower figure shows Cell viability detected by EdU. Fluorescence microscopy images of EdU incorporation, illustrating the results. *P<0.05, **P<0.01, *t* test.

**Figure 5:**
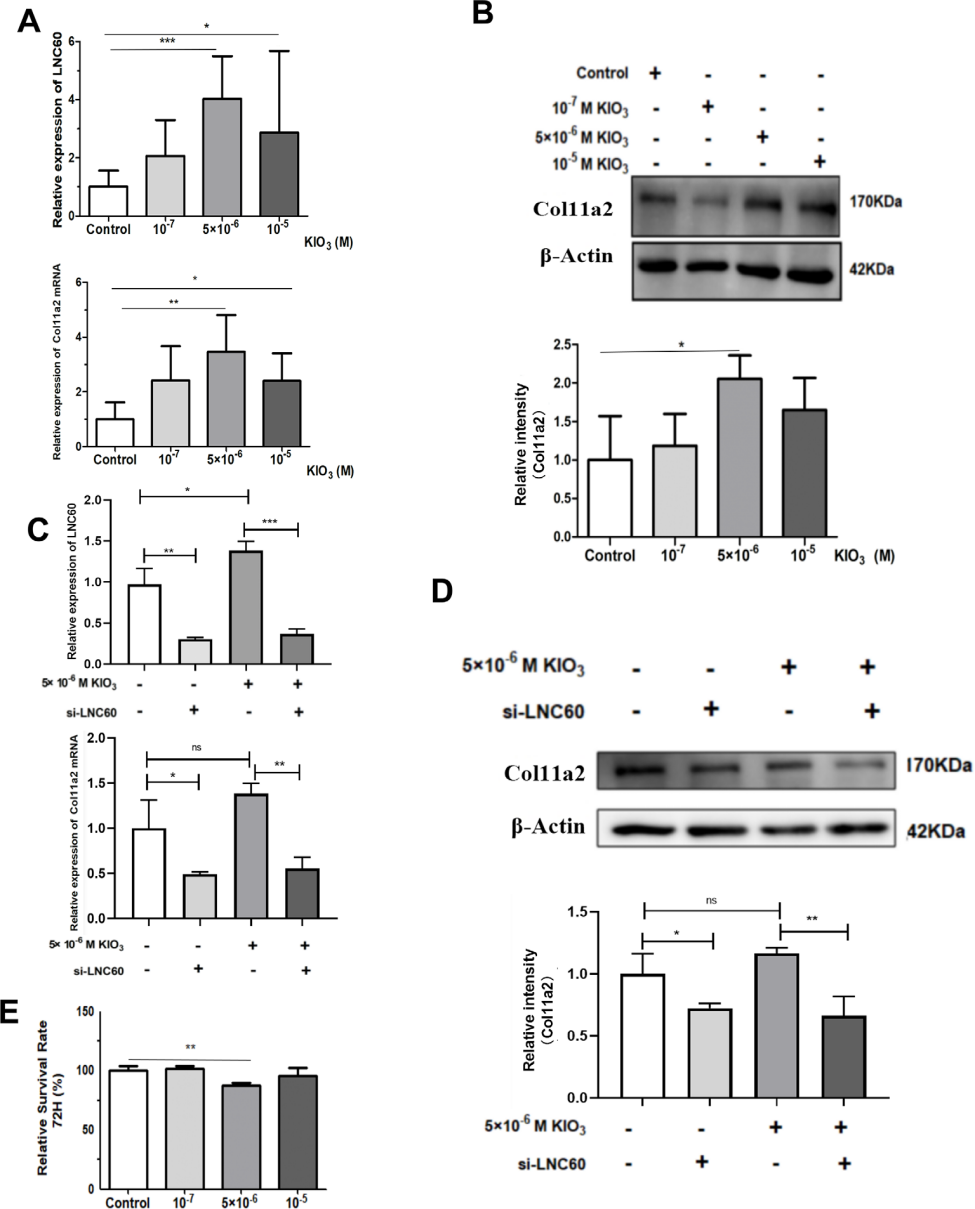
LNC60-Col11a2 axis in Nthy-ori-1 cells. **(A)** After treatment with 10^5^ and 5×10^6^ KIO_3_ for 72 h, qPCR showed that LNC60 and Col11a2 mRNA levels were significantly increased compared with those in the control group. **(B)** With 5×10^6^ KIO_3_ treated for 72 h, Wb shows Col11a2 protein level was significant increased compared with the Control group. **(C)** After siLNC60, qPCR showed that LNC60 and Col11a2 were significantly decreased compared with those in the NC group. **(D)** After siLNC60, Wb showed that the Col11a2 protein level was significantly decreased compared with that in the NC group. **(E)** After siLNC60, the CCK8 assay showed a significant cell viability inhibition in the 5×10^6^ M KIO_3_ treatment group at 72 hours. *P<0.05, **P<0.01, ***P<0.001, *t test*.

### The expression of LNC60 and Col11a2 were induced by high-iodine treatment in Nthy-ori-3 cells

CCK8 and EdU assays were used to evaluate the cell viability of Nthy-ori-3 cells treated with different iodine concentrations (0, 10^-7^ M, 5×10^-6^ M, 10^-5^ M) for 72 hours. The CCK8 results showed that 5×10^-6^ M iodine promote Nthy-ori-3 cells viability (**Figure 4A2**). EdU results showed that 5×10^-6^ M and 10^-5^ M KIO_3_ promote Nthy-ori-3 cells viability (**Figures 4B1–2**). Then, Nthyroi-3 cells were treated with different iodine concentrations for 72 hours. qRT-PCR results showed that compared to the control group, the expression of LNC60 and Col11a2 was significantly increased in the 10^-5^ M and the 5×10^-6^ M treatment groups (**Figure 5A**). WB results showed that compared to the control group, the 5×10^-6^ M treatment group had significantly increased Col11a2 protein expression (**Figure 5B**).

### The expression of Col11a2 was regulated by LNC60

After 72 hours transfection of siRNA-LNC60, the LNC60 expression level was significantly reduced (knockdown rate was 70.60%). After 72 hours of culture with 5×10^-6^ M KIO_3_, the LNC60 expression level was significantly reduced in the siRNA-LNC60 group (knockdown rate was 74.10%) compared to the siRNA-NC group. qPCR and WB results showed that after knocking down LNC60, the expression of Col11a2 before and after iodine treatment was significantly reduced. Regardless of whether iodine treatment was administered, the siRNA-LNC60 group exhibited significantly lower Col11a2 expression than the siRNA-NC group, and iodine treatment may enhance the Col11a2 expression inhibition (**Figures 5C, D**). Furthermore, after knocking down siRNA-LNC60, the cell viability decreased compared to that of the control group after treatment with 5×10^-6^ M KIO_3_ (**Figure 5E**).

### The expression levels of Col11a2 mRNA and LNC60 were increased in peripheral blood of nodular goiter patients from high water iodine areas of China

**Figure 6:**
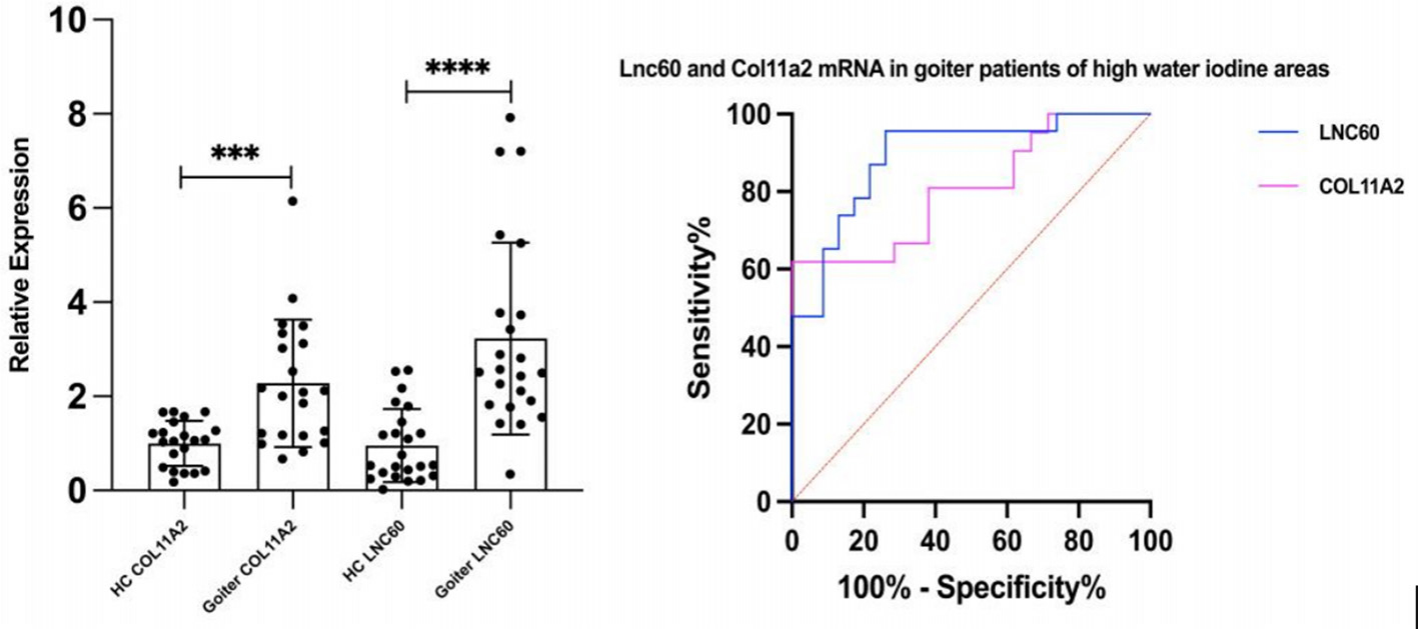
The expression of LNC60 and Col11a2 mRNA in the peripheral blood of Goiter patients and health control populations. ^***^ *P<*0.001, ****P<0.0001, *t* test.

In this study, 26 patients with nodular goiter and 26 healthy control subjects were selected in high- iodine areas (water iodine content >100 μg/L). There were 5 males and 21 females in both groups, with ages of 44.80 ± 9.68 years and 45.80 ± 8.26 years, respectively. The thyroid volume of the patient group was 31.62 ± 7.95 ml, and that of the control group was 9.37 ± 3.22 ml. There was a significant difference in thyroid volume between the two groups (P<0.05). qPCR results showed that compared to the healthy control population, the LNC60 and Col11a2 mRNA levels in whole blood of thyroid goiter patients were significantly elevated (*P*<0.001 and *P*<0.01). Receiver operating characteristic (ROC) curve results showed that AUC of LNC60 was 89.97% (95% CI: 78.991%-98.96%), *P*<0.001. AUC of Col11a2 was 84.85% (95% CI: 72.75%-96.41%), *P*<0.001. Therefore, LNC60 and Col11a2 have high diagnostic values for nodular goiter patients from high water iodine areas of China (**Figure 6**).

## Discussion

Iodine excess always induced colloid goiter, also known as diffuse goiter. In this study, thyroid in the 10-week high-iodine group exhibited pathological features such as enlarged follicles, increased colloid, flattened cell nuclei, and inflammatory responses. The 20-week high-iodine thyroid goiter group not only had more severe above pathological features, but also had other more severe pathological structural changes, including thinning or loss of the basement membrane, overlapping cell nuclei, and other features. Therefore, the model of goiter is successfully constructed. Lei et al. found that the 12,000 μg/L KIO3 group showed a decrease in mitochondria at seven months under TEM microscopy (21). Our TEM results showed that in addition to all the ultrastructural changes of the 10-week group, such as mitochondrial swelling, endoplasmic reticulum widening and cell nucleus flattening, microvilli ciliation, cell pyrosis and even cell necrosis occurred in the 20-week group of hyperiodinated goiter. However, there is no mitochondrial loss. The reason may be that our iodine excess is light.

Goiter is not necessarily accompanied by abnormal thyroid function. Previous studies found that mice drinking excessive iodine water (1500-3000μg/L KIO_3_) for 10 weeks to 7 months could cause colloid goiter, but there were serious thyroid function abnormalities at the same time (21–23). In order to better simulate the formation process of simple goiter caused by iodine excess in the population, to avoid severe thyroid function abnormality, this study chose 300uμg/L KIO_3_ with 10 weeks and 20 weeks, respectively. The results showed that the TSH in the 300μg/L high-iodine drinking water group at 10 weeks did not change, but that in the 20-week high-iodine drinking water group was significantly increased (*P*<0.05). However, there was no statistical difference in serum FT4 level compared with the control group. This suggests that 20 weeks of high iodine feeding may cause subclinical hypothyroidism in mice. The results of this experiment are similar to those of previous studies. Liu Shujun et al. fed Kunming mice with 300μg/L high-iodine drinking water for 10 and 20 weeks, and found that the TSH level of mice was higher than that of the control group, but it is not statistically significant (24). It was also found that TT3,TT4 and TSH levels did not change significantly after feeding mice water with 300μg/L and 600ug/L iodine for three months (25). Rats may tolerate higher doses, and Teng et al. found that feeding Wistar rats with three and six times the dose of high iodine for 24 weeks did not affect TSH levels (26). Shan et al. fed Wistar rats with 50 times the dose of iodine for 12 weeks and 24 weeks, and found that although the serum TSH of the rats was increased, T3 and T4 were still not affected by high iodine (27). Therefore, whether iodine can cause goiter accompanied by abnormal thyroid function is related to iodine dosage and treatment time. The model established in this study will be more helpful to explore the mechanisms of goiter.

In recent years, many lncRNAs have been found to be closely related to thyroid cancer (28). However, there has been no report on lncRNAs related to goiter. In this study, we found that LNC89 was significantly upregulated in both 10-week-old and 20-week-old goiter mice, indicating its close association with goiter and suggesting its potential importance in the early diagnosis and screening of goiter. Human LNC60 is homolog to LNC89. So far, there is no report about LNC89 or LNC60. The cell viability increased in Nthy-ori-3 cells treated with 10^-6^ M KIO_3_, while LNC60 was significantly upregulated. Knocking down LNC60 resulted in Nthy-ori-3 cell viability inhibition with 10^-6^ M KIO_3_ treatment, suggesting an association between LNC60 and cell viability. In this study, the expression of LNC60 in the whole blood of nodular goiter patients in high-iodine areas significantly increased. So that, LNC89/LNC60 may be related to iodine induced goiter or nodular goiter.

Constructing a lncRNA−mRNA coexpression network is a powerful method for inferring the function of lncRNAs (14). In this study, a new LNC89/LNC60-Col11a2 target was constructed. The qPCR results demonstrated that Col11a2 was significantly upregulated in the 10-week and 20-week thyroid goiter mice, suggesting its association with goiter. When Nthy-ori-3 cells were treated with 10^-6^ M KIO_3_, cell viability improved, accompanied by increased Col11a2 expression. Inhibiting LNC60 reduced Col11a2 expression, particularly after iodine treatment, highlighting a more pronounced inhibitory effect. The expression of Col11a2 in the whole blood of thyroid goiter patients in high-iodine areas significantly increased, too. Cooperate with the LNC89/LNC60 results, we proved that LNC89/LNC60-Col11a2 may be related to iodine induced goiter or nodular goiter.

Multiple studies have shown that collagen proteins, including Col11a2, are related to growth, differentiation, tissue remodeling, wound healing, and cell proliferation. Collagen genes are known to be associated with thyroid diseases, such as Col1a1, which is significantly upregulated in papillary thyroid carcinoma (PTC). Analysis of TCGA data has indicated that Col1a1 plays a crucial role in PTC recurrence and adverse prognosis. Col11a2 is a collagen gene whose function loss is associated with various syndromes involving deafness (29). We know that deaf is one of symptoms included in cretinism, which is related to iodine deficiency. This gene is also closely related to multiple fibrotic diseases, such as cardiac fibrosis, liver fibrosis, and lung fibrosis, and is a relatively common fibrotic disease gene (30–32). As shown above, we found Col11a2 up regulated in goiter mice groups. We also found a significant increase in Col11a2 expression in patients with nodular goiter. A small amount of fibrosis can be seen in the thyroid tissue of patients with high iodine-induced goiter (33, 34). Therefore, Col11a2 involved goiter progress may be related to fibrosis and iodine regulation.

However, this study has some limitations. First, cell function experiments need to be perfected, including overexpression of lnc60, over expression and knockdown of Col11a2. Second, the sample size of goiter patients is limited and should be expanded, and include multi-centers and areas with different iodine nutrition levels, making the lnc60−Col11a2 axis more meaningful. Thus, future research should enrich functional experiments and expand population studies to elucidate the mechanisms and assess the potential role of the LNC60-Col11a2 axis as diagnostic and prognostic markers for goiter including nodular goiter.

In summary, this study provides preliminary evidence for the involvement of the novel LNC89/LNC60-Col11a2 axis in the development of iodine-induced thyroid goiter, shedding light on early diagnostic biomarkers and therapeutic targets for thyroid goiter. More, this study not only enriches the lncRNA library, but also provides a new regulatory mechanism for Col11a2-related other diseases.

## Data Availability

The datasets presented in this study can be found in online repositories. The names of the repository/repositories and accession number(s) can be found below: PRJNA1005249 (SRA).
