# Supplementary material for: A new LNC89/LNC60-Col11a2 axis revealed by whole-transcriptome analysis may be associated with goiters related to excess iodine nutrition
Source: Front Endocrinol (Lausanne). 2024 Oct 31;15:1407859. doi: 10.3389/fendo.2024.1407859 (PMC11570895; doi:10.3389/fendo.2024.1407859)
Supplement: Supplementary file 1 [file Table1.docx]

Supplementary Table 1 Primers

| NONMMUG013401.2-F | CCTCCCAAGTGCTGGGATTA |
| --- | --- |
| NONMMUG013401.2-R | CATTGCAGCCCAAACTCAGA |
| NONMMUG011211.2-F | CACCAGAAGAGGGCATCAGA |
| NONMMUG011211.2-R | GCTGGAAAGATGGCTCAGTG |
| NONMMUG027871.2-F | GCTTCCCATGCCCTTTGTAG |
| NONMMUG027871.2-R | CTGGGATTAAAGGCCTGTGC |
| NONMMUG029425.2-F | GTCTTGCTGTGTGCTCTCTC |
| NONMMUG029425.2-R | ACACGCATTCTCTCAAGCAC |
| NONMMUG019446.2-F | CTGGAGCAATGGCTCAACAC |
| NONMMUG019446.2-R | GAGCTACTGGCAGGTGTGAA |
| NONMMUG018089.2-F | ATTCTCTCCTTCCCTCCT |
| NONMMUG018089.2-R | GGTCTCCTGATCCCTCAT |
| Mouse Col11a2-F | CCCTGTCCGCTTTCTCTATGA |
| Mouse Col11a2-R | GGTAACTCGCTTCTTACAGTCC |
| Mouse β-Actin-F | GGCTGTATTCCCCTCCATCG |
| Mouse β-Actin-R | CCAGTTGGTAACAATGCCATGT |
| NONHSAT207060.1-F | AAAAGGAGGGAGTTACAC |
| NONHSAT207060.1-R | CCTACACGAACACAGACA |
| Human Col11a2-F | TCTTTGGTGCCCGTATTCTGG |
| Human Col11a2-R | GGAACCGCTCATTGCCAAT |
| Human β-Actin-F | GGGAAATCGTGCGTGACATT |
| Humanβ-Actin-R | GGAACCGCTCATTGCCAAT |
